# Visual attention toward emotional stimuli: Anxiety symptoms correspond to distinct gaze patterns

**DOI:** 10.1371/journal.pone.0250176

**Published:** 2021-05-13

**Authors:** Lauren A. Rutter, Daniel J. Norton, Timothy A. Brown

**Affiliations:** 1 Department of Psychological and Brain Sciences, Indiana University-Bloomington, Bloomington, Indiana, United States of America; 2 Department of Psychology, Gordon College, Wenham, Massachusetts, United States of America; 3 Department of Psychological and Brain Sciences, Boston University, Boston, Massachusetts, United States of America; Bournemouth University, UNITED KINGDOM

## Abstract

Decades of research have established a link between emotional disorders and attentional biases for emotional stimuli, but the relationship between symptom severity and visual attention is still not fully understood. Depression has been associated with increased attention towards dysphoric stimuli and decreased attention on positive stimuli (“negativity bias”), and some studies have also shown this trend in anxiety disorders. We examined eye fixation variables in 47 participants with emotional disorders completing an emotion recognition task. Results showed that depression severity was not associated with increased fixations on dysphoric stimuli, however, higher levels of generalized anxiety predicted increased fixations in the mouth region of sad and happy faces. Higher levels of social interaction anxiety predicted reduced fixations in the eye region of happy faces. While we did not replicate the negativity bias that has been shown in prior studies, our sample was highly comorbid, indicating the need to consider comorbidity, disorder severity, and the task itself when conducting research on visual attention in clinical samples. Additionally, more attention should be paid to the mouth region of emotional faces, as it may provide more specific information regarding the visual processing of emotions.

## Introduction

A large body of research has demonstrated attentional biases when viewing emotional stimuli in individuals with emotional disorders. Eye tracking technology provides a direct and continuous measure of overt visual attention, and has been used as an important metric in emotion recognition (ER) tasks. In a meta-analytic review of eye-tracking and affective disorders, results showed that when presented with an array of at least two stimuli, depressed individuals were characterized by reduced orienting to positive stimuli, reduced maintenance of gaze on positive stimuli, and increased gaze on dysphoric stimuli [1, 2]. Individuals with depression may voluntarily gaze less at positive stimuli because they are less sensitive to the pleasantness of it, reducing their incentive to maintain gaze. Research has suggested that the strong anhedonic bias in depression is not unique to depression, but an aspect of low positive affect more generally [1, 3, 4].

The relationship between anxiety-related processes and visual attention has been the subject of many research studies (e.g., [5–7]), but there is still not a consensus on how different types of anxiety symptoms and their severity impact visual attention. In general, individuals with anxiety disorders show increased vigilance to threat during free viewing and visual search and difficulty disengaging from threat during visual search but not during free viewing tasks relative to controls [1]. Recent work has examined the relations between worry, rumination, and visual attention, and found that self-reported rumination is linked to greater attention to sad stimuli [8, 9] but worry, compared with rumination, leads to relative avoidance of positive information [10]. Whether this is true across anxiety disorders, representing a transdiagnostic process, remains to be tested.

Relative to common anxiety disorders like generalized anxiety disorder, more is known about visual attention in social anxiety disorder, where eye gaze is likely to be more relevant to the maintenance of symptoms (e.g., [11]; see Staugaard [12] for a review). Individuals with social anxiety disorder may be more sensitive to detecting threat, and may misinterpret faces as threatening when they make quick judgments [13, 14]. In a dot-probe task with emotional faces, Schofield and colleagues [14] found that social anxiety was associated with attention to emotional (rather than neutral) faces over time, and difficulty engaging attention from angry faces. Heuer and colleagues [13] administered a morphed faces task (a task involving watching a series of computer-morphed faces that change slowly from a neutral to a fully emotional expression) to socially anxious individuals and non-anxious controls with time pressure (restricted viewing task, RVT) or with unlimited viewing of the faces (free viewing task, FVT). Participants with high levels of social anxiety demonstrated a threat bias (disgust interpreted as contempt) in the RVT, contrasting with the non-anxious control group’s positive bias (disgust interpreted as happy). No group differences were found in the FVT. Thus, time spent on faces and the pressure of making an accurate judgment in ER tasks may be linked with levels of anxiety and social anxiety. Additionally, more recent research has indicated that performance-based social anxiety specifically, when compared to panic disorder with agoraphobia, appears to present a sustained bias for vigilant attention to aversive facial expressions, but for the most severe social anxiety, patients show an opposing avoidance of aversive facial expressions [15]. This indicates that the severity as well as type of social anxiety play a role in emotional face perception and visual attention.

While much of the literature has looked at the eye region as an area of interest in ER some researchers have defined areas in the lower face (mouth) as particularly important in recognizing emotion. It is important to consider areas of interest within the entire face, not just the eye region, when examining emotion recognition in clinical groups, as there may be differences in visual attention patterns based on disorder status or severity. For example, in highly socially anxious individuals, looking at the mouth may be more comforting than staring into eyes. Wong and colleagues [16] examined patterns of visual scanning as predictors of emotion identification in older adults and younger adults. Older adults who made more fixations in the top halves of faces were more accurate at identifying emotions than those who made more fixations in the lower halves of faces. However, for the emotion of disgust in particular, older adult participants were more accurate when they fixated on the lower half of the face. In the younger adult group, analyses revealed a significant positive correlation between emotion-identification accuracy and difference scores computed on the number of fixations made to the top half versus bottom halves of faces [16]. In another study, individuals with high-functioning autism fixated more in the mouth region of faces even when the faces were inverted [17]). These authors suggest that abnormal gaze in individuals with high-functioning autism is driven by an impaired top-down strategy for allocating visual attention. Accordingly, it seems necessary to examine eye gaze patterns for each of the emotional faces when gauging where participants are attending.

The current study examines clinical factors as dimensional variables within a patient sample that is highly comorbid on depression and anxiety diagnoses. While prior research has compared visual attention patterns in depressed and anxious groups to healthy controls, the comparison of visual attention patterns in clinical groups is lacking. Moreover, while the emotion recognition literature has established impairments in emotion recognition in samples with anxiety and mood disorders compared to the general population, we do not yet know if a reason for these deficits is based in differences in time spent on certain regions of interest on an emotional face (eyes vs. mouth) or due to other reasons. Previous group-based comparisons may have led to inconsistent or weaker ER results because individual differences in symptom severity were collapsed into a binary variable. Our approach takes into consideration the heterogeneity of depression symptoms, the severity of anxiety symptoms, and their comorbidity [18]. In addition, we were equipped to attempt to disentangle social anxiety factors from other forms of anxiety.

In order to clarify the relationship between visual attention and anxiety and mood symptom severity, we used an entirely clinical sample with anxiety and mood disorder diagnoses. We had four hypotheses, each based on prior research. First, we expected there would be more fixations on negative faces in depressed participants compared to anxious based on the prior finding that depressed individuals attend more to dysphoric stimuli (e.g., [1]). Of note, this hypothesis was based on a finding comparing depressed individuals to healthy control subjects, and it remains to be tested to see if the anxiety group differs significantly from the depressed group. Next, we hypothesized that higher levels of depression would be associated with more fixations on sad faces based on the established finding that depressed individuals show a specific attentional bias to sad, but not angry or threatening facial expressions (e.g., [8, 19]). Third, we predicted that higher anxiety scores would predict more fixations in threatening faces (fear) based on the prior finding that social anxiety is associated with difficulty disengaging from threat (e.g., [14, 20]), specifically the indirect or ambiguous threat of a fearful face. Of note, we used an emotion recognition task that was different from the findings for which we based our prediction, as many tests of threat-bias include a paired face forced choice paradigm, and we used a dynamic emotional face task. Additionally, there is a body of work that shows that angry faces can elicit a threat-related attentional bias in anxious individuals (see Bar-Haim et al, 2007) [21], so we examined visual attention differences in angry faces (direct threat) in addition to fearful faces (indirect threat) based on level of anxiety. Last, we wanted to explore if there was reduced fixation on positive faces in high levels of worry and anxiety. Based on the finding that worry, compared to rumination, leads to relative avoidance of positive information ([10] we expected that higher anxiety severity would predict reduced fixation on happy faces.

## Materials and methods

### Participants

This study was approved by the Boston University Institutional Review Board. Written informed consent was obtained from all individual participants included in the study.

Eligible participants were males and females >= 18 years of age with a principal diagnosis of an anxiety or mood disorder who presented for assessment and treatment at the Center for Anxiety and Related Disorders at Boston University. Participants were recruited for a study examining the effect of intranasal oxytocin on emotion recognition performance and visual attention (see Rutter et al., 2019a) [22]. Exclusionary criteria were: (1) current delusions or hallucinations, (2) current suicidal or homicidal risk meriting intervention, (3) two or more hospitalizations in the last 5 years for severe psychopathology (psychosis, suicide attempts), (4) not fluent English speakers (those unable to complete a phone screen and clinical interview in English), (5) pregnancy, (6) a current or past autism spectrum disorder diagnosis, (7) regular smokers (smoking more than 15 cigarettes/day), or consumers of non-prescription or illicit drugs (except for oral contraceptives), (8) major sensory impairment and/or visual acuity score (binocular) worse than 20/40, (9) those who are currently experiencing a respiratory illness requiring medication (i.e., allergy, cold, or flu symptoms), and (10) those who are suffering from a chronic medical condition (i.e., heart disease, uncontrolled hypertension, myocardial infarction, cardiac arrhythmia, kidney or liver disease, vascular disease, epilepsy, migraine, asthma, nephritis, diabetes or another endocrine disease, frequent or unexplained fainting, stroke, aneurism or brain hemorrhage, or other neurological illness).

Individuals with a variety of anxiety and mood disorders were recruited for this study (N = 60). We recruited participants with the following clinical diagnoses into our depression (n = 30) cohort: major depressive disorder, persistent depressive disorder, other specified depressive disorder, and unspecified depressive disorder. We recruited individuals with the following diagnoses into our anxiety (n = 30) cohort: panic, agoraphobia, specific phobia, separation anxiety, social anxiety, generalized anxiety, other specified and unspecified anxiety, obsessive-compulsive, and posttraumatic stress disorders. While OCD and PTSD are not technically anxiety disorders as defined by DSM-5, we included them in this group because of their similarities to other anxiety disorders (and differences from depressive disorders). Of note, participants in the anxiety cohort could not have a current clinical mood disorder, but comorbid anxiety disorders were allowed into the depressed group. Thus, the depression group represented higher levels of mood and anxiety disorder comorbidity, while the anxiety group was purposely designed to filter out mood disorders. Individuals in both the depressed group and anxiety group may have been assigned multiple anxiety disorder diagnoses, but no one in the anxiety group had a clinical level of mood disorder psychopathology present at the time of their diagnostic interview. The main difference between the depression group and anxiety group was the presence of a unipolar depressive disorder in the depressed group and the absence of a unipolar depressive disorder in the anxiety group.

The average age was 27.21 (SD = 9.73, range = 18—65). The sample was predominantly female (n = 28; 59.57%), Caucasian (n = 34; 72.3%; Asian = 10.1%; African American = 6.4%, Other/not reported = 4.2%), and non-Hispanic (n = 36; 76%). Visual acuity was calculated while participants used corrective eyewear. The average visual acuity score was above 20/20 (M = 1.19), and ranged from 0.58 to 1.34 (SD = .17). Based on exclusionary criteria of vision being 20/40 or better, all participants were eligible.

The sample breakdown of principal diagnoses was as follows: generalized anxiety disorder (23.3%), social phobia (21.7%), coprincipal diagnosis (10%), specific phobia (10%), PDD (8.3%), obsessive-compulsive disorder (8.3%), MDD (6.7%), body dysmorphic disorder (3.3%), other specified anxiety disorder (3.3%), panic disorder (1.7%), other specified obsessive-compulsive and related disorder (1.7%), other specified trauma/stressor-related disorder (1.7%). Of note, 70% in the depressed group had a principal or co-principal anxiety disorder (see [22]).

### Measures

**Anxiety and Related Disorders Interview Schedule for DSM-5** (**ADIS-5** [23]). The ADIS-5 is a semi-structured interview designed to establish a diagnosis of DSM-5 anxiety, mood, somatoform, obsessive-compulsive, trauma, and substance use disorders, and to screen for other disorders (e.g., psychotic disorders). The ADIS-5 was administered by trained Ph.D.-level psychologists and advanced doctoral students in clinical psychology with extensive training to meet strict certification criteria (see [23] for details). We used the ADIS to determine participants’ primary diagnoses and place them into the anxious or depressed groups.

**Beck Depression Inventory-II** (**BDI**; [24]). The BDI-II is a widely used 21-item self-report measure of severity of depressive symptoms. Higher scores represent more severe depression. The BDI-II has been shown to have strong psychometric properties in outpatient samples [25].

**Beck Anxiety Inventory** (**BAI**; [26]). The BAI is a widely used 21-item self-report measure of severity of anxiety symptoms. Higher scores represent more severe anxiety. The BAI has been shown to have strong psychometric properties in outpatient samples [26].

**Social Interaction Anxiety Scale** (**SIAS**; [27]). The SIAS is 20-item self-report measure of anxiety surrounding social interactions. Higher scores indicate more severe social interaction anxiety [27]. The SIAS is shown to be a useful measure in screening, designing personalized treatments, and evaluating outcomes of treatments for social anxiety [28]

**Emotion recognition task** (“**facial morphing**”). This task entails watching computer-morphed faces that change slowly from a neutral (0% emotionality) to a fully emotional expression (100%). Stimuli faces were taken from Ekman and Friesen’s (1976) Series of Facial Affect [29]. Using Matlab software, each face was presented for 500ms. The black-and-white face images were approximately 12.25 x 9 cm in size, presented on the middle of the screen on black background of a Hewlett Packard FP2141sb 21” CRT monitor. Participants were presented with a neutral face (0% emotionality), which progressed in 2% increments toward 100% emotionality. Each increment of emotionality, or frame, was displayed for 500 ms, with every fifth frame jittered to be 1, 2, 3, 4, or 5 times the normal 500 ms length, to weaken the relationship between time and emotional intensity. After responding to two practice trials, participants were shown 40 morphed sequences (male and female actor expressing angry, happy, fear, and sad emotion five times each) of the faces in random order [22]. These 40 test trials were each comprised of a dynamic sequence of a face changing from a neutral expression to an emotional expression.

Participants were asked to press a keyboard key as soon as they detected an identifiable emotional expression. Pressing the key cleared the screen and prompted participants to identify the face as expressing happiness, sadness, fear, or anger. Accuracy was recorded for each emotion type, as was the emotional intensity of the morphed expression at the time of the keyboard press. Possible intensities ranged from 0 (neutral) to 100 (fully morphed emotion). Higher intensity scores indicate that participants required greater emotion to identify the emotion type, and were slower to respond. Intensity scores were only calculated for accurate trials only. Trials where participants pressed the space bar to select face type at 0% intensity (i.e., neutral) were not scored as accurate or incorrect, and intensity scores were not calculated, because this response style indicates that the participants were holding down the space bar when the trial began and never saw the emotional face stimulus.

### Eye tracking

#### Hardware

An Applied Science Laboratories Eye-Trac 6 eye-tracking system was used to record the position of eye gaze throughout the task (see [22] for more specific procedures). The system has maximum accuracy of 0.5 degrees of visual angle, with a resolution of 0.25 degrees. The temporal resolution of the camera was set at 120 Hz. A chin rest was used to reduce head movement. After adjusting the camera of the eyetracker to be centered on the participant’s dominant eye, a short calibration sequence was administered whereby the participant looked at 9 points across the display monitor. After this sequence, the eye-tracking system was able to accurately and continually calculate the participant’s point of gaze relative to the display.

#### Data reduction

After the initial 9-point calibration, the accuracy of the system was checked at the beginning of each trial for a period of 1500 milliseconds during which the participant fixated centrally (i.e., fixation cross). An average of the calibration checks across the 40 trials was used to refine the original calibration. Individual trials where the fixation during this 1500 millisecond period were aberrant were excluded. Data from each trial were only analyzed if valid eye data were collected for at least 50% of the duration of that trial. Data from particular subjects were excluded from analysis if that participant lost more than 50% of their trials. Of the 60 participants recruited, a total of 13 were lost due to this system. This system of data reduction eliminates erroneous and missing data due to issues with calibration, mechanical problems, and experimenter error [22]. Our final sample size was 47 participants, with 22 in the Depressed group and 25 in the Anxious group.

#### Eye fixation variables

Eye movement parameters reflecting the topographical characteristics of scanning behavior were the proportion of fixations on previously defined regions of interest of the face: entire face, eye region, and mouth region. For each trial, we calculated average percent fixation data in the eye region relative to data in the face, and mouth region relative to face [30]. For this reason, we did not remove eye and mouth regions when examining fixations in the face, as data was calculated based on proportion of fixations relative to the entire face. Fixations were defined as the participant keeping their gaze within a 1 degree area for at least 100 milliseconds. Thus, from the reduced eye data we calculated proportion of fixation data relative to other data (i.e., sum of time length of each fixation during the trial divided by length of the trial) in each region of interest (i.e., face, eyes, mouth) for each face type (i.e., happy, sad, angry, fearful). For example, “Eye/face” is the proportion of fixation data captured in the eye region relative to the proportion of data captured in the face region, and “Mouth/face” is the proportion of fixation data captured in the mouth region relative to proportion of data captured in the face region [30]. Table 1 presents proportion of fixation data relative to other data in each region of interest (i.e., face, eyes, mouth) for each face type (i.e., happy, sad, angry, fearful). We also took the average of all emotional conditions to calculate a grand mean of face, eye, and mouth proportions for the fixation data. Since, for the majority of each trial, the emotional faces are indistinguishable from each other (in fact, the subject ends the trial immediately once they are able to discern what emotion the face bears), this represents an additional primary outcome measure, see Table 2.

**Table 1:** Summary of visual attention by diagnostic group status.

	Depressed (*n* = 22)		Anxious only (*n* = 25)	
	Mean	*SD*	Mean	*SD*
Sad expressions				
Face/screen	.90	.11	.93	.11
Eye/face	.49	.23	.60	.16
Mouth/face	.21	.14	.19	.14
Happy expressions				
Face/screen	.90	.13	.94	.09
Eye/face	.48	.22	.56	.16
Mouth/face	.24	.16	.26	.18
Fearful expressions				
Face/screen	.86	.20	.92	.12
Eye/face	.50	.25	.60	.18
Mouth/face	.21	.16	.18	.15
Angry expressions				
Face/screen	.90	.10	.93	.10
Eye/face	.48	.22	.56	.18
Mouth/face	.24	.15	.20	.15

*Note*. Face/Screen = the proportion of fixation data captured in the face region relative to data captured outside of the face, on the computer screen. Eye/face = the proportion of fixation data captured in the eye region relative to the proportion of data captured in the face region. Mouth/face = the proportion of fixation data captured in the mouth region relative to proportion of data captured in the face region.

**Table 2:** Correlations between fixations and symptom severity scores.

Variable	BDI	BAI	SIAS
Proportion of fixations on sad face	-.06	.26	-.07
Proportion of fixations on sad eyes	-.21	-.03	-.32*
Proportion of fixations on sad mouth	.16	.38*	.18
Proportion of fixations on happy face	-.07	.15	-.10
Proportion of fixations on happy eyes	-.23	-.16	-.41**
Proportion of fixations on happy mouth	.13	.34*	.25
Proportion of fixations on fearful face	-.22	.25	-.07
Proportion of fixations on fearful eyes	-.26	-.01	-.34*
Proportion of fixations on fearful mouth	.13	.30*	.26
Proportion of fixations on angry face	-.17	-.09	-.06
Proportion of fixations on angry eyes	-.21	-.19	-.28
Proportion of fixations on angry mouth	.13	.28	.26
Grand proportion of fixations on faces	-.16	.19	-.09
Grand proportion of fixations on eyes	-.24	-.10	-.36*
Grand proportion of fixations on mouths	.15	.34*	.25

*Note*.

* *p* <.05,

** *p* <.01. BDI = Beck Depression Inventory; BAI = Beck Anxiety Inventory; SIAS = Social Interaction Anxiety Scale.

#### Data analyses

RStudio was used to conduct analyses. We conducted a series of regression analyses to test our hypotheses and control for covariates. We used a conservative Bonferroni correction of p <.017 to account for multiple comparisons. For Hypotheses 1 we conducted t-tests to compare eye fixation data between depressed and anxious groups. For Hypothesis 2, we regressed depression severity (BDI-II) onto fixations in the eye region of sad faces. For Hypothesis 3, we regressed anxiety severity (BAI and SIAS) onto proportion of fixations on the entire face, the eye region, and the mouth region of fearful faces. For Hypothesis 4, we regressed anxiety severity onto fixations in regions of interest in happy faces. As mentioned, these data are part of a larger randomized controlled study [22] where half of the participants received oxytocin and half received placebo before completing the emotion recognition task.

We used R pwr package to verify that our sample was adequately powered to detect significance after lost participants due to calibration errors and missing eye data. Our final sample size of 47 provided adequate power (*β* = .80) to detect effects of medium to large size (f^2^ = .25; *α* = .05) using multiple regression (Cohen, 1992) and t-tests (d = .58, *α* = .05).

## Results

Descriptive statistics are presented in Tables 1 and 2. With regard to emotion recognition performance, average accuracy scores for all faces in our sample was.93 (SD = .06, range .78 –1.0)[happy = .999, angry = .84, fearful = .93, sad = .97) while intensity scores averaged 40.57 (SD = 9.10) and ranged from 21.03—70.77)[happy = 29.40, angry = 46.25, fearful = 43.61, sad = 44.83]. There were no differences in accuracy or intensity scores between the Depressed and Anxious groups (p >.05). The differences between ER accuracy and intensity scores based on drug status (oxytocin vs. placebo) between Depressed and Anxious groups is the subject of prior work [22]. The average BAI score in our sample was 18.20 (SD = 9.60, range = 0-44), indicating moderate anxiety. The average BDI score in our sample was 19.37 (SD = 10.99, range = 0—41), indicating mild depression. The average SIAS score in our sample was 35.42 (SD = 19.64, range = 0-77), indicating moderate social interaction anxiety.

**Fig 1:**
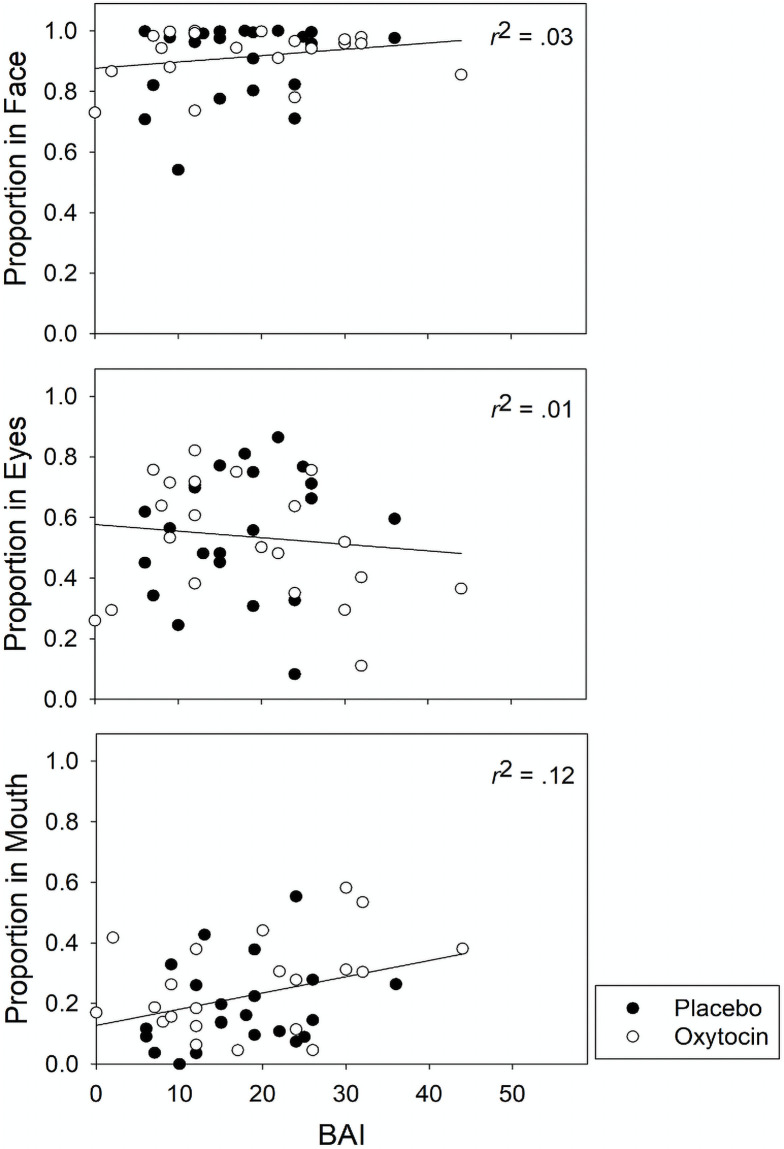
Proportion of fixations in regions of interest by Beck Anxiety Inventory (BAI) scores and oxytocin/placebo status. The black circle represents participants who received placebo. The white circle represents participants who received oxytocin. BAI = Beck Anxiety Inventory.

Before proceeding with any analyses, we tested to see that there were no significant differences in eye gaze patterns between participants who received oxytocin (n = 22) and those who received placebo (n = 25) in our sample using a series of Welch Two Sample-tests. Fig 1 displays the proportion of fixations in the face, eye, and mouth regions for all emotional faces (happy, sad, angry, and fearful), plotted by anxiety severity (BAI) and placebo and oxytocin status. There were no significant differences in proportion of fixations in any region of the face for any emotion between the oxytocin and placebo groups (p range.11 in mouth region of happy faces to.80 in sad faces). We then tested the relationship between a mood disorder and fixations on negative faces, expecting more fixations on sad, angry, and fearful faces if the participant was depressed vs. anxious using a series of Welch Two Sample-tests (Anxious only n = 25; Depressed n = 22). None of these were significant (p range.21 for proportion in fearful faces to.44 for proportion fixations in sad faces).

We expected that higher depression scores based on BDI-II would be associated with more fixations on sad faces. Results showed that higher depression scores were not significantly associated with increased fixations in sad faces overall (p = .7), or in the eye (p = .17) or mouth regions (p = .30) of sad faces. Thus, contrary to our hypothesis, depression severity did not predict fixations on sad faces. To follow up on this unexpected finding, we tested the relationship between depression severity and fixations on angry, happy, and fearful faces, and found that depression severity did not predict any fixations on negative (angry, fearful) or positive (happy) faces (p >.05).

Next, we tested the hypothesis that higher anxiety scores would be correlated with more fixations on threat, i.e., fearful faces. Using fearful faces, we examined the relationship between proportion of fixations on the entire face, the eye region, and the mouth region using regression. Results showed that higher anxiety scores based on BAI were associated with more fixations in the mouth region of fearful faces (R^2^ = .09, F (1, 41) = 4.18, *β* = .30, p <.05), although the effect size was small (f^2^ = .10), and did not survive Bonferroni correction. The relationship between level of anxiety and proportion of fixations in the entire face and eye region of fearful faces was not significant (p >.05). We then examined the connection between levels of social anxiety, based on the SIAS, and fixations in regions of interest. Results showed that higher levels of social anxiety were associated with decreased attention to the eye region of fearful faces (R^2^ = .12, F (1, 41) = 5.48, *β* = -.34, p <.05, f^2^ = .14). This did not remain significant after Bonferroni correction. Higher levels of social anxiety did not predict fixations in the mouth or entire face (p >.05), see Table 2.

To test our last hypothesis, we examined the relationship between anxiety severity and eye gaze fixation using multiple regression, with the hypothesis that higher anxiety severity (based on BAI) would be associated with reduced fixations in happy faces. Higher anxiety scores did not predict reduced fixations on happy faces overall, or in the eye region of happy faces. There were, however, significant differences in attention to the mouth region for happiness and other emotional faces, described below.

To examine the relationship between BAI and eye gaze fixations, we controlled for depression status, thinking that the relationship between eye tracking and anxiety may be changed by the presence of mood disorder. Additionally, although we had already established that there was no main effect for oxytocin on eye gaze variables, we controlled for oxytocin here to confirm that there was not an interaction effect of drug and anxiety severity. Results showed that higher anxiety (BAI) was associated with increased fixations in the mouth region of happy faces (R^2^ = .18, F (3, 39) = 2.86, *β* = .38, p<.05) and mouth region of sad faces (R^2^ = .20, F (3, 39) = 3.28, *β* = .39, p<.05), with small effects range f^2^ = .22—.25, which survived Bonferroni correction. We also explored the relationship between social anxiety severity and visual attention, and found that increased social interaction anxiety, as measured by the SIAS, was associated with significantly decreased fixations in the eye region of happy faces (R^2^ = .23, F (3, 39) = 3.81, *β* = -.38, f^2^ = .30), which was significant after Bonferroni correction. Additionally, increased social anxiety severity was associated with decreased fixations in the eye region of fearful faces (R^2^ = .19, F (3, 39) = 3.11, *β* = -.31, p<.05), f^2^ = .23), which did not remain significant after accounting for multiple comparisons using Bonferroni methods. The relationship between social anxiety severity and attention to the eye region of sad faces approached significance (R^2^ = .18, F (3, 39) = 2.82, *β* = -.29, p = .051). When oxytocin was removed as a covariate, this finding was significant: social anxiety severity predicted decreased attention to the eye region of sad faces (R^2^ = .14, F (2, 40) = 3.39, *β* = -.29, p<.05). Interestingly, this indicates that oxytocin may have a particular effect on visual attention in individuals with higher levels of social anxiety, even though oxytocin failed to produce a main effect on visual attention overall. This finding is consistent with the research using oxytocin to enhance treatment of social anxiety disorder [31]

## Discussion

While a large body of literature has shown that emotional disorders are characterized by attentional biases for emotional stimuli, to our knowledge, this study was the first to examine the impact of symptom severity on attention to specific regions of interrest in emotional faces in a comorbid and transdiagnostic entirely clinical sample. This advance from the existing research is notable, given high rates of comorbidity between anxiety and mood disorders [32] and lack of consensus in the field as to whether biases in attention represent a general or specific deficit in emotion recognition [33, 34].

## Comorbidity

Our results showed that having a depression diagnosis vs. anxiety only diagnosis did not significantly impact visual attention: there were no differences between attention to sad, happy, angry, or fearful faces based on depression status. Next, results showed that higher anxiety scores based on the BAI were associated with more fixations in the mouth region of happy faces and sad faces, but not angry or fearful faces. In contrast, higher SIAS scores did not predict visual attention to the mouth region. Higher SIAS scores were associated with decreased fixations in the eye region of happy faces.

### Depression and negativity bias

Because depression has been associated with increased attention to negative stimuli, one might have expected some bias in our study on sad, fearful, or angry faces particularly, as compared with happy faces. Indeed, we made hypotheses that depression severity would be associated with a negativity bias. However, when carefully considering the nature of prior paradigms in contrast to the current one, it makes sense that we did not in fact find that depression severity correlated with any measure of visual attention. Prior studies showed a bias for depressed individuals to gaze at negative stimuli comparatively more than controls when presented with a negative and non-negative stimulus simultaneously [8]. Our paradigm only had a single stimulus. The only observable bias was the proportion of gaze allocated to the various “zones” of eyes versus face and mouth regions. Given that we do not possess clear hypotheses for why an eye or a mouth on a face of a given emotion would be systematically considered more negative, we do not believe that a negative gaze bias in depression would reveal itself at all in our paradigm.

### Anxiety, but not social anxiety, and attention to the mouth

This study explored how visual attention was related to both generalized anxiety severity and social interaction anxiety severity in a transdiagnostic sample. More research has established a connection between social anxiety and visual attention than generalized anxiety and visual attention, due to the clear connections between social anxiety disorder and eye contact. Thus, one might have expected an especially strong relation between social anxiety and eye tracking variables for two reasons. First, social anxiety is associated with biased gaze patterns in several studies [11, 12, 14]. Second, the social nature of the face stimuli might call for biases such as averting gaze from the eyes. However, we found that general anxiety, as measured by the BAI, was correlated with mouth gaze, but social anxiety, as measured by the SIAS, was not. The reasons for this are unclear, but one scenario might be that a desire for sureity which is linked to anxiety generally, and not social anxiety specifically. All our stimuli were ambiguous at the outset, since they started as neutral faces. Overall, the visual shift in mouth movements for emotional signals (such as the corners rising or falling) are more overt and stronger than those in the eyes, which rely on higher spatial frequency information such as wrinkles beginning to form. Individuals with high levels of anxiety and low comfort with uncertainty may prefer, relatively, to view the mouth as a sure thing, at least on some trials. Of course, that factor would be at work along with others in determining scanning behavior, which the high-anxiety individuals in this study did mostly in a fashion similar to those with low-anxiety.

Our study was not the first to show the importance of examining the mouth region of emotional faces. Indeed, early research [35] showed that both upper and lower regions are involved in processing visual cues of different face types, and more recent work has suggested that the mouth is the most important cue for static and dynamic faces [36]. Prior work in nonclinical samples has shown a bias towards examining the mouth region of happy faces compared to fearful or neutral expressions, where there is a consistent pattern of preferentially scanning the eye region [37] Reduced attention to the eye region has been linked to amygdala hypoactivation and genetic differences in the 5-HTTLPR promoter polymorphism [38], but we did not examine these variables in our study, and instead focused on symptom severity.

There are many ways that our study expands on the literature and several reasons for the differences in our results from previous studies. First, our sample is entirely clinical without a healthy control group. To date, much of the research on visual attention and emotion recognition compares disordered cohorts to control groups, showing more deficits in the disordered sample. Our study represents an advance through an entirely clinical sample, allowing us to explore differences based on severity. However, substantial heterogeneity, comorbidity, and individual differences within diagnostic groups may provide explanations of nonsignificant effects visual attention in clinical samples. Additionally, because we examined relationships dimensionally within a clinical group, it is possible that our current dimensional approach was unsuccessful is because our sample did not have severe enough levels of anxiety and depression and to detect differences [18]. For example, comparing a clinical group to a healthy control group is more powered to detect differences than a dimensional approach such as ours, especially if the range of anxiety and depression symptoms is not wide enough to adequately represent more severe clinical cases.

While our diverse clinical sample was a major strength of our research design, some limitations should be considered. Due to the lack of research in this area, and the failure to standardize ER and eye tracking procedures, the generalizability of findings is limited. Additionally, we could not use 13 out of 60 participants’ eye data due to mechanical or calibration errors. Eye tracking results should be interpreted with caution. Using more fine-grained eye tracking methods or advanced technologies is a recommended direction for future research. Furthermore, while significant, our effect sizes were small and some did not remain significant when corrected for multiple comparisons with a conservative Bonferroni estimate. Additionally, it is not clear if the ER task we used is the best way to measure visual attention towards emotional stimuli. In fact, despite the advantage in ecological validity to use dynamic faces over static ones, some recent studies have used negative and positive images to measure visual attention in clinical cohorts, including the recent Lewis et al [10] study that influenced our study hypotheses. Moreover, despite their potential advantage in ecological validity, traditional ER tasks are riddled with measurement constraints such as ceiling effects for happiness and response bias [39–41].

### Conclusions

In sum, we found that higher anxiety scores on the BAI were associated with more fixations in the mouth region of happy and sad faces. These results indicate the value of looking at dynamic visual stimuli, as the same patterns were not observed in all regions, and the importance of considering severity of symptoms in addition to diagnostic labels. Our findings indicate the need for future studies in clinical samples, with additional variables, including personality factors [42], that can explore mechanisms for differences in visual attention and ER. Moreover, future work should continue to recruit transdiagnostic and comorbid samples to better understand the complex relationship between emotion recognition, visual attention, and depression and anxiety. Future research should continue to examine if there is a bias in attending to positive emotions that could be responsible for maintenance of negative affect, and how to correct this in treatment. If attentional biases could be corrected, there are important clinical implications for symptom improvement and disorder recovery.
